# Survey of dentin sialophosphoprotein and its cognate matrix metalloproteinase‐20 in human cancers

**DOI:** 10.1002/cam4.2117

**Published:** 2019-04-01

**Authors:** Jaya Aseervatham, Saxena Geetu, Charles C. Anunobi, Komal Koli, Kalu U. E. Ogbureke

**Affiliations:** ^1^ Department of Diagnostic and Biomedical Sciences University of Texas School of Dentistry at Houston Houston Texas; ^2^ Department of Anatomic and Molecular Pathology, College of Medicine University of Lagos Lagos Nigeria

**Keywords:** cancer, dentin sialophosphoprotein, immunofluorescence, immunohistochemistry, in situ proximity ligation assay, MMP20, SIBLING‐MMP interaction, tissue microarrays

## Abstract

**Background:**

Matrix metalloproteinases‐20 (MMP20) expression is widely regarded as tooth specific, with expression limited to dental hard tissues. Recently, we reported MMP20 expression and interaction with dentin sialophosphoprotein (DSPP), a member of the Small Integrin Binding Ligand N‐linked Glycoproteins (SIBLINGs), in human oral squamous cell carcinoma (OSCC) and dysplastic oral premalignant lesions (OPLs), suggesting a role for MMP20‐DSPP interaction in oral carcinogenesis.

**Methods:**

This study aimed to survey the expression of MMP20 and its cognate DSPP partner in the breast, colon, prostate, thyroid, and cervical neoplasms. Using commercially available tissue microarrays (TMAs) and cell lines, we performed immunohistochemistry, immunofluorescence, proximity ligation assay, and western blot experiments to determine the expressions of MMP20 and DSPP in the breast, colon, prostate, thyroid, cervical neoplasms, and their normal counterparts.

**Results:**

Significantly high expression levels of MMP20 and DSPP were observed in the malignant breast, colon, prostate, thyroid, and cervical neoplasms compared with their benign and normal counterparts. Furthermore, MMP20 levels increased with advanced stages of colon and thyroid cancers. DSPP expression increased significantly with tumor stage in all cancers examined.

**Conclusions:**

The co‐localization and potential MMP20‐DSPP interaction previously reported in oral cancers are present in other cancers. These results suggest MMP20‐DSPP pairing as a potential marker of disease activity in some epithelial cancers with diagnostic and prognostic implications.

## 1 INTRODUCTION

Matrix metalloproteinase‐20 (MMP20) is a member of the matrix metalloproteinase family, a group of zinc‐dependent metallopeptidases, involved in extracellular matrix remodeling. In humans, the MMP20 gene is localized on chromosome 11q22.3‐q23 with 10 coding exons.^1^ Consistent with the characteristic domain structure of most MMPs, MMP20 exhibits a signal peptide, a propeptide necessary to maintain enzyme latency, a zinc‐binding site within a catalytic domain, and a hinge region linking the catalytic domain to a C‐terminal hemoplexin‐like domain.^2^, ^3^, ^4^, ^5^, ^6^ Until recently, MMP20 expression was thought to be restricted to developing dental hard tissue.^7^, ^8^, ^9^ Specifically, it has been shown that MMP20 is required during the initial developmental stages of enamel and loss of its activity results in qualitative defects in enamel formation.^10^

Dentin sialophosphoprotein (DSPP) is a member of the Small Integrin Binding Ligand, N‐linked Glycoprotein (SIBLING) family. Along with other members of the family, the DSPP gene is clustered on a narrow region of approximately 375,000 base pairs on chromosome 4 in human and chromosome 5 in mice. Other members of the family are bone sialoprotein (BSP), osteopontin (OPN), dentin matrix protein 1 (DMP1), and matrix extracellular phosphoglycoprotein (MEPE).^11^ DSPP is hardly ever isolated as an intact protein because soon after its synthesis it is proteolytically processed into dentin phosphoprotein (DPP), dentin sialoprotein (DSP), and dentin glycoprotein (DGP) in developing teeth by MMP2 and MMP20.^12^ DPP, initiates and modulates the formation and growth of hydroxyapatite crystals during dentin formation^13^, ^14^ while DSP regulates the initiation of dentin mineralization.^15^ Furthermore, recently published reports indicate that mouse carboxy‐terminal DSP enhanced the attachment, migration, proliferation, and differentiation of human periodontal ligament stem cells, regulating gene expression of teeth and bone markers, growth factors, and transcription.^16^ The precise role of DGP is yet to be fully understood.

Reports of the upregulation of DSPP and other members of the SIBLING family with their cognate MMP partners in human cancers, along with their diagnostic and prognostic significance, have been published.^17^, ^18^, ^19^, ^20^, ^21^, ^22^, ^23^, ^24^ Recently, we reported the expression of MMP20 in human oral squamous cell carcinoma (OSCC), and in metabolically active duct epithelial tissues of the salivary gland and kidney nephron.^7^, ^8^, ^9^ Our reports also established MMP20‐DSPP co‐localization and interaction in these biologic systems.^7^, ^8^, ^9^ Using human tissue microarrays (TMAs), tissue lysates, and cell lines, we investigated the expression and interaction of MMP20 with DSPP in the breast, colon, thyroid, prostate, and cervix neoplasms in our study. Our study is based on the hypothesis that DSPP and its cognate MMP20 partner are upregulated in some human epithelial neoplasm where co‐localization and interaction, seen in OSCC, may also take place.

## 2 MATERIALS AND METHODS

### 2.1 Human tissue microarrays (TMAs)

Human TMAs were purchased from Protein Biotechnologies (San Diego, CA). Different TMAs used in the study include human breast cancer (cat # TMA‐1001), cervical cancer (cat # TMA‐2220 and TMA‐2218), thyroid cancer (cat # TMA‐1502), prostate cancer (cat # TMA‐2208), and colon cancer (cat # TMA‐1414).

### 2.2 Cells, culture conditions, and antibodies

Epithelial HPV‐16 E6/E7 transformed cells (Ect1/E6E7), cervical carcinoma cell line DoTc2, and squamous cell carcinoma (SCC) cell line SiHa were purchased from ATCC and grown in keratinocyte‐serum free media (Gibco), ATCC‐formulated DMEM, and ATCC formulated‐EMEM, respectively. Breast cancer cell lines, MCF12F and MCF7, were purchased from ATCC and grown in complete DMEM and Ham's F12 growth medium containing 20 ng/mL epidermal growth factor (sigma), 100 ng/mL cholera toxin (sigma), 0.01 mg/mL human insulin (sigma), 500 ng/mL hydrocortisone (sigma), and 5% chelex‐treated horse serum for MCF12F. For MCF7, the medium was ATCC formulated EMEM to which 0.01 mg/mL human recombinant insulin and fetal bovine serum were added to a final concentration of 10%. Human primary prostate epithelial cell line (HPEpiC) was purchased from ATCC and was grown in prostate epithelial basal medium (ATCC) to which the following supplements were added. l‐Glutamine, Extract P, epinephrine, rhTGF‐α, hydrocortisone, rh‐insulin, and apo‐transferrin. The cells were maintained with 5% CO_2_ under standard conditions. Antibodies used in this study were anti‐MMP20 (Bioss; 1:200 for IHC, IF and PLA; 1:1000 for WB) and anti‐DSPP (Santa Cruz Biotechnologies; 1:200 for IHC, IF, and PLA; 1:1000 for WB). The use of these commercially available antibodies has been published in previous studies.^25^, ^26^, ^27^, ^28^

### 2.3 Western blot (WB)

Western blot was performed on commercially available tissue lysates and whole cell extracts from cell lines to determine the expression of MMP20 and DSPP. Tissue lysates for the normal and cancer breast (cat# T2‐001, T2‐020, T2‐046, T2‐050, and T2‐054), colon (cat# T7‐002, T7‐004, and T7‐008), cervix (cat# T4‐022; T4‐023; T4‐024, and T4‐025), prostate (cat# T3‐001, T3‐007, T3‐010, T3‐032, T3‐036, and T3‐037), and thyroid (cat# T16‐003, T16‐008, T16‐023, T16‐024, and T16‐033) tissues were purchased from Protein Biotechnologies (San Diego, CA). Whole cell extracts from different cell lines to the breast (MCF7 and MCF12), colon (CaCo2, Colo320DM and HT29), cervix (Ect1/E6E7, SiHa and DoTc2) and prostate (HEpiC, PC3, and LNCap) were made using RIPA buffer containing protease and phosphatase inhibitors. Total amount of protein was quantitated, and equal amounts loaded and resolved with 4%‐20% Criterion TGX (cat # 567‐1094, BioRad, Hercules, CA) SDS‐polyacrylamide gel electrophoresis. This was followed by electrophoretic transfer using a Trans‐Blot Turbo Transfer System (cat# 170‐4155EDU, BioRad, Hercules, CA) to a low‐fluorescence PVDF membrane (cat# 20130403, BioRad, Hercules). Assay was performed in triplicate (N = 3). Proteins were detected using respective primary and secondary antibodies, and imaged using a LiCor Odyssey scanner. Band intensity was normalized using *β*‐actin, and quantified.

### 2.4 Immunohistochemistry (IHC)

Standard immunoperoxidase techniques were carried out on ~5 μm sections of formalin‐fixed, paraffin‐embedded TMAs of the breast, prostate, colon, and cervix neoplasms, and their normal counterparts as previously described.^7^, ^8^, ^9^ Some of the tissue arrays contained sections of nonneoplastic inflammatory pathology. The Intellipath FLX automated system (Biocare Medical, Concord, CA), which included the Intellipath FLX universal HRP‐detection kit (IPK5011, Biocare Medical) was used following the manufacturer's instructions. Briefly, TMA sections were deparaffinized in three series of xylene and rehydrated through series of ethanol (100%, 95%, 70%) and water. Antigen retrieval was performed before endogenous peroxidase quenching was carried out for 10 minutes. Thereafter, sections were treated with background punisher (catalog # BP974H; Biocare Medical) for 20 minutes in order to reduce nonspecific binding. Sections were then loaded onto the preprogrammed and timed autostainer for sequential primary (DSPP/MMP20) and secondary antibody incubation. Chromogens were detected with either 3,3′‐diaminobenzidine (BDB2004), or Warp Red (WR806H) for single‐stained sections, and with both chromogens for double labeling. Sections were counterstained with hematoxylin for 10 seconds. Negative control consisted of sections treated with Universal Negative Control Serum (NC498, Biocare Medical). Representative photographs were captured with Nikon DS‐U3 digital camera fixed to Eclipse Ni‐E microscope with accompanying NIS Elements AR software (Nikon, Melville, NY).

### 2.5 Scoring of IHC results

We have previously reported a semiquantitative scoring method for immunostains on tissue sections. Briefly, immunoreactivity for MMP20/DSPP was scored as “negative” (0, not detectable/faint staining <10% of tumor tumor/parenchymal cell); “1” (10%‐50% immunoreactive tumor cells); “2” (50%‐75% immunoreactive tumor/parenchymal cells); and “3” (widely/intensely expressed in tumor/parenchymal cells).

### 2.6 Immunofluorescence (IF)

Cells plated on coverslips in a 6‐well dish overnight, were washed with 1XPBS and fixed in 4% paraformaldehyde (PFA, Sigma‐Aldrich; cat# P6148) for 20 minutes, before permeabilization in 0.1% triton X‐100 for 10 minutes. Cells were then treated with blocking buffer (1XPBS, 1% goat serum) for 1 hour at room temperature, followed by overnight incubation at 4°C with primary antibodies to DSPP and MMP20 diluted in blocking buffer. Coverslips were washed 3X with 1XPBS and incubated with secondary antibodies for 1 hour before mounting with Prolong Gold anti‐fade reagent with DAPI (cat # P36931, Life technologies Grand Island, NY).

### 2.7 In situ proximity ligation assay (iPLA)

In situ proximity ligation assay (iPLA) was used to verify specific cellular interactions of MMP20 with DSPP in the fixed human breast, colon, prostate, cervix, and thyroid tissue sections. Prior to iPLA, paraffin‐embedded tissue sections were deparaffinized and antigen retrieved. iPLA was performed using the HRP‐detection kit from Olink Bioscience according to the manufacturer's protocol (cat # DUO92012; Sigma‐Aldrich, St. Louis, MO). Briefly, tissue sections were incubated with primary anti‐MMP20 polyclonal (rabbit) antibody and anti‐DSPP monoclonal (mouse) antibody or with normal rabbit/mouse IgG. They were then incubated with corresponding secondary antibodies conjugated to oligonucleotides PLA probes (MINUS and PLUS) for 1 hour at 37°C. Rolling circle amplification (RCA) was performed using T4‐ligase as described by the manufacturer (Olink Bioscience St Louis, MO). HRP oligonucleotides (cat # DUO92012; Sigma‐Aldrich, St. Louis, MO) were used to detect RCA products in tissues sections. The protein interactions were observed as brown punctate signals and were captured using Nikon Eclipse Ni‐E microscope and NIS Elements AR software (Nikon, Melville, NY).

### 2.8 Statistical analysis

Results were analyzed using either Student's *t* test or one‐way analysis of variance (ANOVA) with subsequent post hoc Tukey's pairwise analysis using GraphPad Prism (version 6, San Diego, CA). The correlation between expression of MMP20 and DSPP in normal and tumors tissues was tested by regression analysis. Data are presented as means ± SEM with statistically significant differences as *P*‐values less than or equal to 0.05.

## 3 RESULTS

We employed immunohistochemistry (IHC), immunofluorescence (IF), and in situ proximity ligation assay (iPLA) to survey five common human neoplasms, along with their normal tissue/nonneoplastic counterpart, for the expression of MMP20 and DSPP, using TMA, lysates, and cell lines. The advent of TMA technology allows for the screening of protein expression by IHC and iPLA on hundreds of tissue samples on a single slide.^29^ In addition, IF and iPLA provided insight into the co‐localization and potential MMP20‐DSPP interactions in normal and neoplastic tissues, and cells.

### 3.1 MMP20 expression in the human breast, colon, prostate, cervix, and thyroid tissues

**Figure 1:**
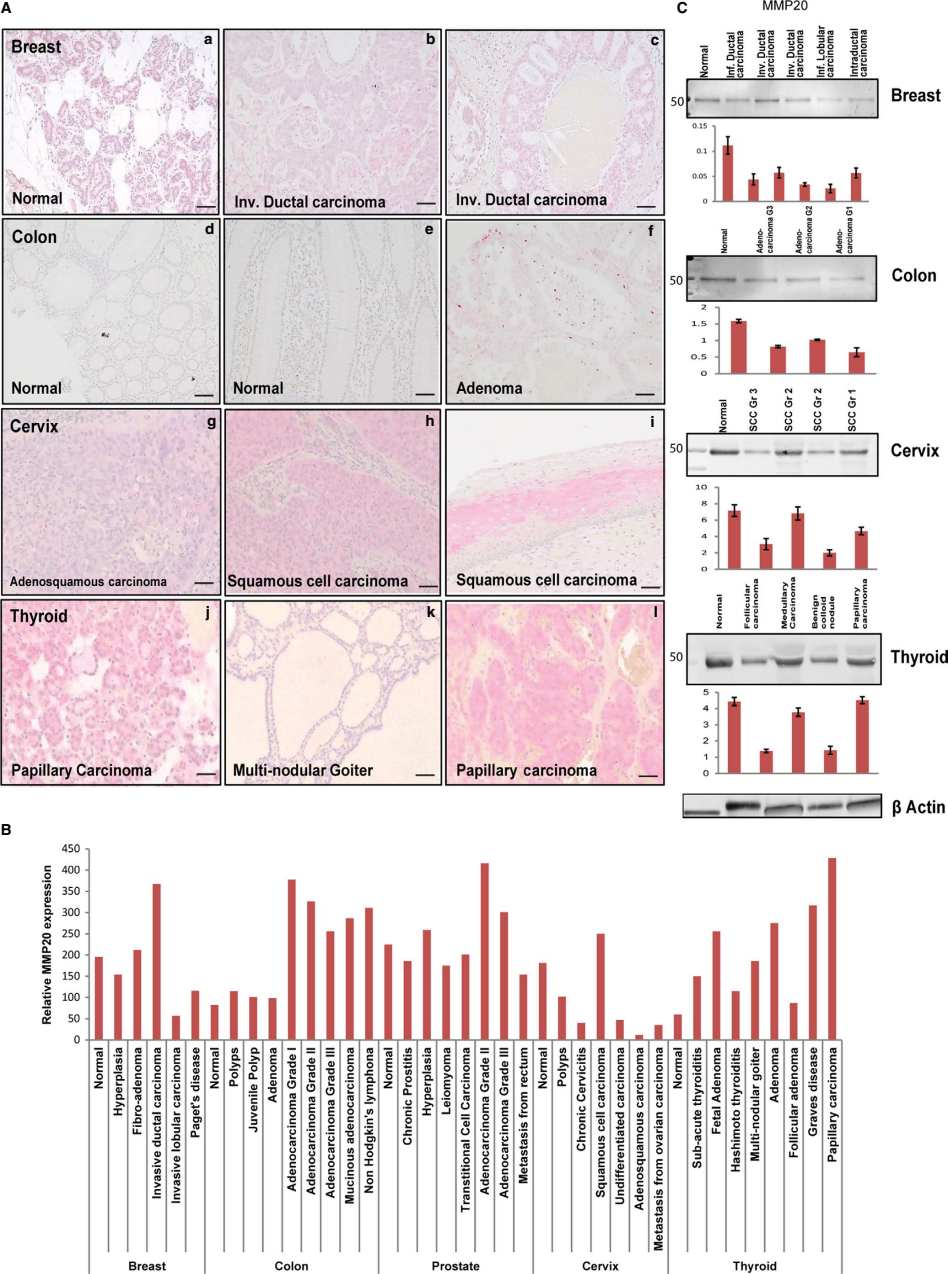
MMP20 is expressed in the normal and neoplastic breast, colon, prostate, cervix, and thyroid tissue sections. A, Immunohistochemistry (IHC) on tissue microarrays (TMA) showing positive immunoreactivity for MMP‐20 in normal and invasive ductal carcinoma of breast tissue (a‐c); normal and adenoma colon tissue (d‐f); normal and squamous cell carcinoma of cervix tissue (g‐i); and, normal and papillary carcinoma of thyroid tissue (j‐l). B, Histogram for quantitative IHC of TMA shows differential expression of MMP‐20 in the normal and different grades of cancer in the breast, colon, prostate, cervix, and thyroid. C, Western blot (WB) and quantitative (fold change) histogram shows significantly high MMP‐20 protein levels in normal and cancer whole tissue lysates from the breast, colon, prostate, cervix, and thyroid tissues. *β*‐actin was used as the normalization control. Values are mean ± SE, n = 3. Data are representative of three independent experiments. Scale bar, 100 μm

As shown in Figure 1, MMP20 was expressed in the normal and neoplastic breast, colon, prostate, cervix, thyroid tissues, and in their respective whole tissue lysate. Representative immunostains (Figure 1A) and quantification (Figure 1B) for MMP20 on tissue sections of the breast, colon, prostate, thyroid, cervical neoplasms, and their normal counterparts are shown, whereas the full TMA immunostain panel for the tissue types is shown in Figure S1 (A‐E; G as IgG control). While detectable levels of MMP20 were present in normal tissues of all five organs, levels were particularly high and comparable to that of some neoplastic counterparts in the breast, prostate, and cervix (Figure 1B). MMP20 levels in normal colon and thyroid remained notably lower than their neoplastic counterparts (Figure 1B).

With respect to breast tissues, levels of MMP20 in invasive ductal carcinoma of the breast were notably higher than that in hyperplasia, invasive lobular carcinoma, and Paget's disease of the breast (Figure 1B). Similarly, MMP20 levels in adenocarcinoma grades I, II, III, mucinous adenocarcinoma, and non‐Hodgkin's lymphoma of the colon were very high compared to normal colon, colonic polyps, and adenomas (Figure 1B). MMP20 levels in adenocarcinoma grade II of the prostate were notably higher than in other prostatic neoplasms, including adenocarcinoma grade III, transitional cell carcinoma, leiomyoma, benign prostatic hyperplasia, and chronic prostatitis (Figure 1B). Except for SCC of the cervix, which showed high levels of MMP20 (comparable to levels in normal tissue counterpart), other neoplasms of the cervix exhibited relatively low levels of MMP20. Although MMP20 levels in inflammatory conditions of the thyroid (subacute thyroiditis, Hashimoto thyroiditis, and Graves disease) and benign thyroid neoplasms (fetal adenoma, adenoma, multinodular goiter) were high, notably higher levels of MMP20 are present in papillary carcinoma (Figure 1B). As shown by western blot analysis of whole tissue lysates, MMP20 levels in the breast, colon, cervix, and thyroid, closely mirror that of their respective tissue section immunostains (Figure 1C).

### 3.2 DSPP expression in the human breast, colon, prostate, cervix, and thyroid tissues

**Figure 2:**
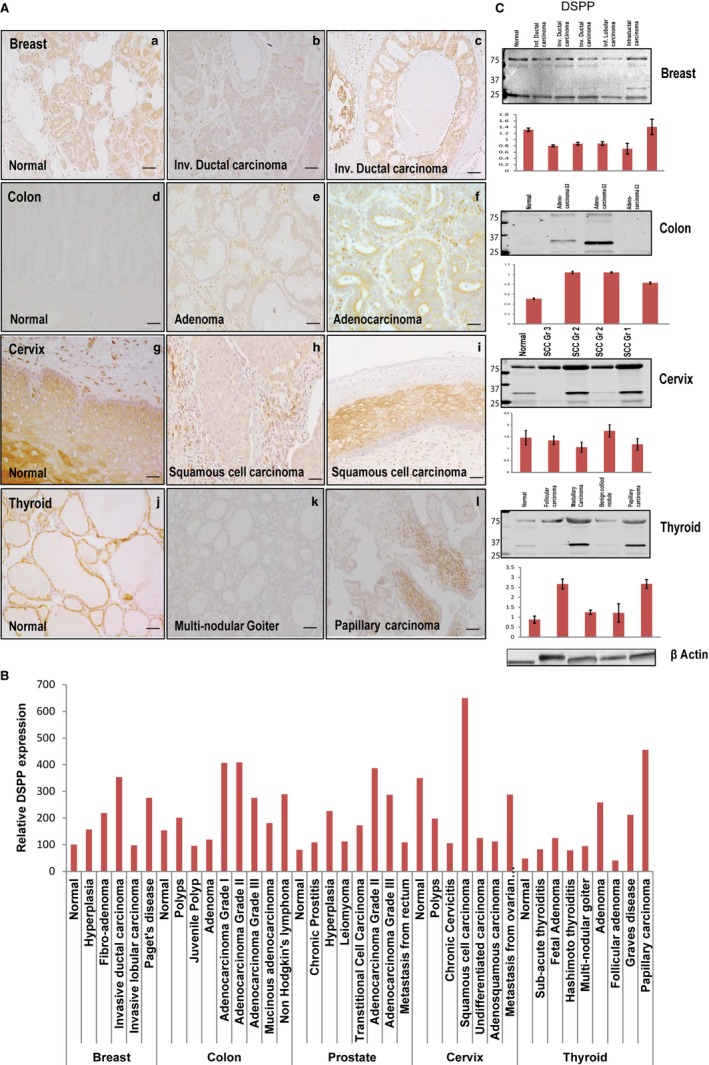
DSPP is expressed in the normal and neoplastic breast, colon, prostate, cervix, and thyroid tissue sections. A, Immunohistochemistry (IHC) on tissue microarrays (TMA) showing positive immunoreactivity for DSPP in normal and invasive ductal carcinoma of breast tissue (a‐c); normal and adenoma colon tissue (d‐f); normal and squamous cell carcinoma of cervix tissue (g‐i); and, normal and papillary carcinoma of thyroid tissue (j‐l). B, Histogram for quantitative IHC of TMA shows differential expression of DSPP in the normal and different grades of cancer in the breast, colon, prostate, cervix, and thyroid. C, Western blot (WB) and quantitative (fold change) histogram shows significantly high DSPP protein levels in normal and cancer whole cell tissue lysates from the breast, colon, prostate, cervix, and thyroid tissues. *β*‐actin was used as the normalization control. Values are mean ± SE, n = 3. Data are representative of two independent experiments. Scale bar, 100 μm

Mirroring MMP20 expression, DSPP levels in all five tissue types and their corresponding whole tissue lysates were expressed in normal and neoplastic counterparts (Figure 2; Figure S2). As shown in representative panels of Figure 2A, positive immunostain for DSPP was evident in the cytoplasm and perinuclear regions of the duct epithelial cells of the breast, colon, and thyroid as well as the cervical mucosal epithelium. DSPP expression levels in the normal breast, colon, and cervix were notably high and comparable to levels in their respective neoplastic counterpart (Figure 2B). However, expression levels in normal prostate and thyroid were relatively lower than in their neoplastic counterpart except for metastatic prostatic adenocarcinoma and follicular adenoma of the thyroid, where DSPP levels compared with their normal counterpart (Figure 2B). Correspondingly, western blot analysis of whole tissue lysates of the normal and neoplastic breast, colon, cervix, and thyroid showed expression levels of DSPP comparable to immunostains on tissue sections (Figure 2C).

### 3.3 MMP20‐DSPP co‐expression in the breast, colon, prostate, cervix, and thyroid

**Figure 3:**
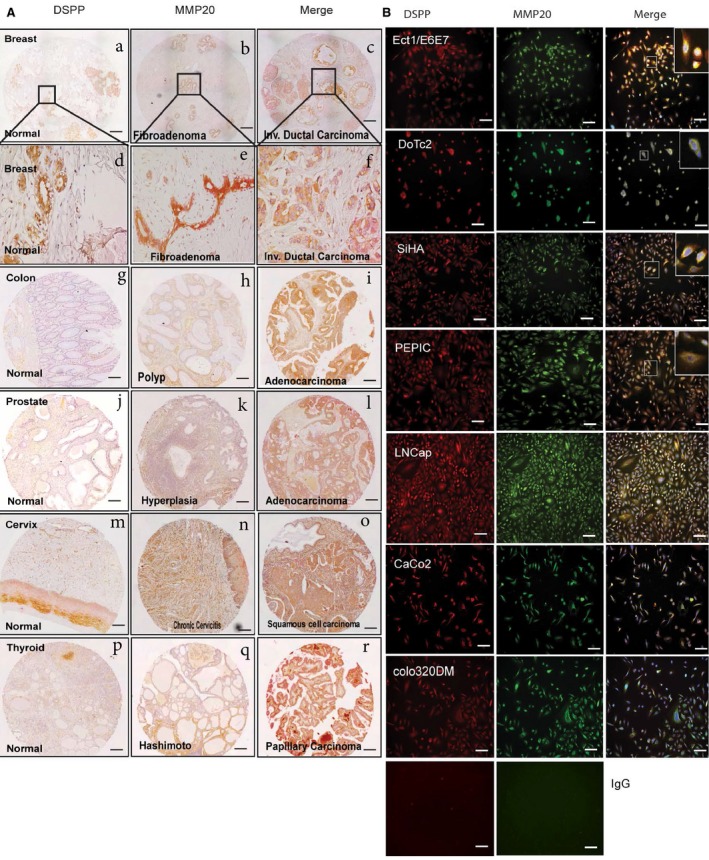
MMP20 is co‐expressed with DSPP in the normal and cancer breast, colon, prostate, cervix, and thyroid tissue microarrays. A, Double‐label IHC shows co‐expression of MMP‐20 (red; alkaline phosphatase) and DSPP (brown; HRP) in normal and invasive ductal carcinoma of breast tissue (a‐f); normal and adenoma colon tissue (g‐i); normal and hyperplasia prostate tissue (j‐l); normal and squamous cell carcinoma of cervix tissue (m‐o); and, normal and papillary carcinoma of thyroid tissue (p‐r). Scale bar, 100 μm. B, Distribution of MMP‐20 (red) and DSPP (green) in normal and cervix, prostate, colon, and breast cancer cell lines. Immunofluorescence (IF) indicates strong signals in ECT1 (normal cervix), DoTC2, and SiHa (cervix cancer cells); LNCap (prostate cancer); and Colo320 (colon cancer cells) in contrast with minimal expression of both MMP‐20 and DSPP in the non‐cancer PEpiC (prostate epithelial cells) and Caco2 (colon cells). The merged insets in ECT1, DoTC2, SiHA, LNCap, and Colo320 evidenced strong co‐localization (yellow) of MMP‐20 with DSPP. The signal was detected using a fluorescent microscope. Scale bar, 100 μm

Our recent report indicates that MMP20 and DSPP co‐express and potentially interact with each other in human OSCC.^9^ In order to investigate the possible co‐expression of MMP20‐DSPP in the normal and neoplastic breast, colon, prostate, cervix, and thyroid tissues, we carried out double‐stain immunohistochemistry and immunofluorescence on TMAs and cell lines, respectively. As illustrated in Figure 3A by double immunostaining on tissue sections, MMP20 and DSPP were co‐expressed in epithelial cells of the breast, colon, prostate, cervix, and thyroid tissues (red‐brown stain; see also Figure S3). Intensity of MMP20‐DSPP stain was as high in normal and inflamed cervical (chronic cervicitis) epithelia as in SCC of the cervix (Figure 3A). Similarly, MMP20‐DSPP staining was of equally high intensity in normal breast ductal epithelial tissue as with fibroadenoma and invasive ductal carcinoma of the breast. On the other hand, MMP20‐DSPP staining intensity was higher in neoplastic counterparts (carcinomas and adenocarcinoma) of the prostate, colon, and thryroid (Figure 3A). Similarly, immunofluorescence (Figure 3B) indicated MMP20‐DSPP co‐expression in cancer cell lines and in normal but transformed cell lines from the colon (Colo320, Caco2), prostate (LNCap, PEpic), and cervix (DoTC2, SiHa, ECT1).

### 3.4 MMP20‐DSPP co‐localization and interaction in multiple human cancers

**Figure 4:**
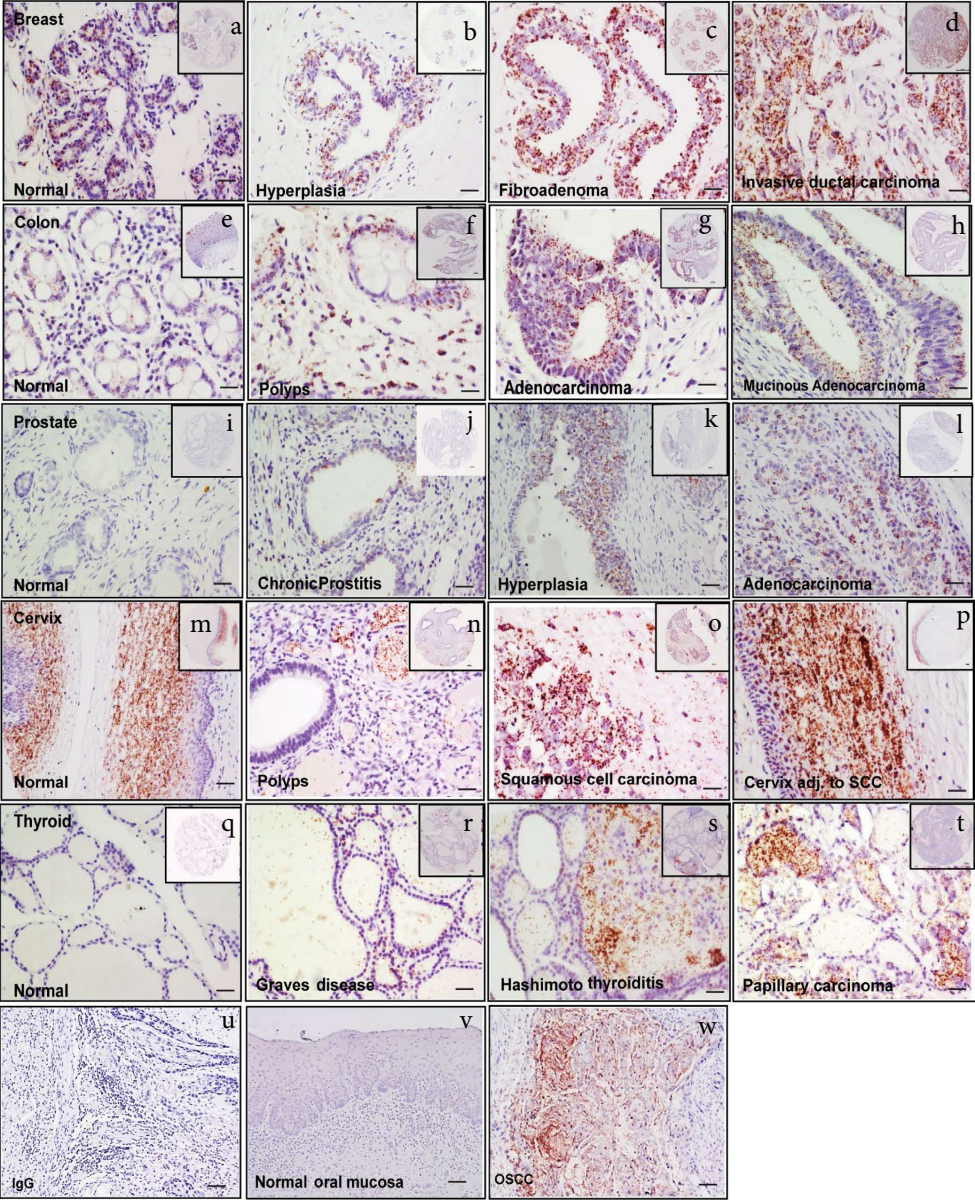
MMP20 interacts with DSPP in the normal and cancer breast, colon, prostate, cervix and thyroid tissue microarrays. Micrographs showing DSPP interaction with MMP‐20 assessed by in situ proximity ligation assay (PLA) in normal and invasive ductal carcinoma of breast tissue (a‐d); normal and adenoma colon tissue (e‐h); normal and hyperplasia prostate tissue (i‐l); normal and squamous cell carcinoma of cervix tissue (m‐p); and, normal and papillary carcinoma of thyroid tissue (q‐t); preimmune IgG negative control (u); normal oral mucosa (tissue) negative control (v); oral squamous cell carcinoma (OSCC) positive control (w). PLA signals (brown dots) were detected using bright field microscopy (20×). Scale bar, 100 μm

Our recent reports show MMP20‐DSPP co‐localization and evidence of interaction in OSCC by iPLA.^9^ As shown in Figure 4 iPLA representative results on TMAs, strong MMP20‐DSPP interaction signals (brown dots; see also Figure S4) were observed in fibroadenomas and invasive ductal carcinomas of the breast compared with hyperplastic and normal breast epithelium. Similarly, although punctate signals were observed in normal and colon polyps, distinctly strong interaction signals characterized adenocarcinomas of the colon. Normal prostate tissues showed complete absence of MMP20‐DSPP interaction signals, punctate signals in chronic prostatitis, and distinctly strong interaction signals in hyperplastic and adenocarcinomas of the prostate. All ranges of cervical epithelia (normal through cancerous) exhibited strong interaction signals for MMP20‐DSPP by iPLA, whereas only Hashimoto thyroiditis and papillary thyroid carcinoma showed strong interaction signals compared to normal thyroid tissue. Negative experimental control is represented by pre‐immune IgG, whereas normal oral mucosa known to show no signal for MMP20‐DSPP interaction was used as tissue negative control. OSCC tissues known to exhibit strong MMP20‐DSPP interaction signals were used as positive control (Figure 4).

## 4 DISCUSSION

Although reports on the expression and potential role of DSPP in the biology of a number of human epithelial cancers have mounted in recent years,^22^, ^23^, ^30^, ^31^, ^32^, ^33^ there only have been sporadic reports on the expression of MMP20 in specific cancers until recently.^9^, ^17^ Our current report therefore represents the first on a survey of the expression profile of MMP20 in an array of various notable human epithelial neoplasms. Our recent report of MMP20‐DSPP expression and potential interaction in OSCC^9^ prompted us to survey the expression profiles of MMP20 and DSPP in the normal and neoplastic breast, colon, prostate, cervix, and thyroid tissues and cell lines.

Fisher et al,^21^ over a decade ago, published the expression profile of other members belonging to the SIBLING family: BSP; OPN, and DMP1 and their cognate MMP partners: MMP2; MMP3; MMP9, respectively, in the breast, uterus, colon, stomach, ovary, lung, rectum, thyroid, and kidney, using cDNA microarrays.^21^ Our current reports include data on the expression profiles of MMP20 and DSPP, as cognate partners, in archived pathologic tissues and cell lines of the breast, colon, prostate, cervix and thyroid neoplasms. Following the trend of our recent report on MMP20 expression in OSCCs,^9^ we also provide cumulative evidence of MMP20‐DSPP co‐localization and interaction in the pathologic breast, colon, prostate, cervix, and thyroid tissue and cell lines, by immunofluorescence and in situ proximity ligation assays.

Our previous reports established the expression of DSPP and its cognate MMP20 partner in duct systems of metabolically active epithelial cells of normal salivary glands, and nephrons.^7^, ^8^ As shown in Figures 1 and 2, the expression of MMP20 and DSPP in the normal breast, colon, and cervical tissues was not merely detectable but in some of the tissue types comparably as high as in their neoplastic counterparts. This suggests that normal physiologic functions of the breast, colon, and cervical epithelia may mirror that of the highly metabolically active duct systems of the salivary gland, nephron, and eccrine sweat ducts. Accordingly, the potential biologic significance of DSPP and MMP20 levels in the breast, colon, and cervical neoplasms is still unclear. In contrast, the absent‐to‐basal level expression of MMP20 and DSPP in normal prostate and thyroid compared to elevated levels in their neoplastic counterpart (see Figures 1 and 2) suggests that the duct epithelial system in normal prostate and the ductless thyroid gland epithelium may be of the less metabolically active type physiologically, mirroring that of metabolically inactive lacrimal duct epithelium.^34^ In this context, the elevated levels of MMP20 and DSPP in prostate and thyroid neoplasms may be of diagnostic and prognostic utility.

In a retrospective study of 73 laryngeal squamous cell carcinoma (LSCC) tissue specimens from patients by Liu et al,^35^ the authors determined, by immunostaining, that MMP20 was overexpressed in LSCC compared to matched normal tissue controls. Significantly, the authors reported expression levels of MMP20 correlation with regional lymph node metastasis (*P* = 0.023). However, there was no significant correlation between MMP20 expression and disease stage (early versus late stage). The authors reasoned that, since MMP20 has a broad substrate specificity that included several proteins (amelogenin, casein, gelatin, fibronectin, collagen IV and XXVIII, laminin‐1 and ‐5, tenascin‐C, and p‐casein), it also may play a role in the pathogenesis of certain cancers.^35^ Our recent report mirrored that of Liu et al, to the extent that MMP20 is also significantly upregulated in human OSCC and oral premalignant lesions (OPLs), compared to normal tissue controls.^9^ However, large, multicentric retrospective and prospective studies of patients will be required to determine any relationship between the expression of MMP20 (and its cognate DSPP) and prognostic indicators such as regional node spread, distant organ metastasis, and tumor recurrence in the oral, breast, colon, prostate, cervix, and thyroid cancers.

Although, the mechanisms of DSPP‐MMP20 interactions in cancers have yet to be fully deciphered, our published data showing a ninefold enrichment of DSPP at the MMP20 promoter in oral cancer cell lines suggest a regulatory role for DSPP in the transcription of MMP20. DSPP, directly and specifically binds MMP20 promoter‐proximal element, and in the process MMP20 peptides (residues 107‐153) within its catalytic domain interact with DSPP peptide (residues 34‐120).^9^ Evidence of the nuclear localization of full‐length DSPP in HEK293, MC3T3‐E1, and DPSC cells have been reported, although the mechanisms of its recruitment to MMP20 promoter remain unclear.^36^ It is however possible that DSPP recruitment to MMP20 promoter is indirect via transcription factors known to interact with MMP20.^9^

Nevertheless, it is speculated that MMP20‐DSPP interactions may involve one or more of the mechanisms already identified for the other SIBLING‐MMP pairing.^37^, ^38^ Alternatively, this may involve a yet to be identified mechanism(s), such as one implicating a yet to be characterized cryptic site within the N‐terminal DSPP sequence that interacts with MMP20.^9^ It is anticipated that such interaction will bridge MMP20 to cell surface receptors such as integrins, to trigger resulting signaling pathways that regulate proliferation, migration, invasion, and metastasis.^9^ For example, earlier reports have shown that DSPP silencing in OSCC cells alters salient tumorigenic hallmarks of oral carcinogenesis such as proliferation, cell cycle activities, invasion, and migration.^32^

In summary, we report MMP20 and DSPP expression and interaction in five common human epithelial neoplasms and their normal tissue counterparts: the breast, colon; cervix; prostate; and thyroid. This report also represents the first on MMP20 expression in these tissues. Whereas, DSPP‐MMP20 expression in the normal and neoplastic breast, colon and cervix epithelial suggests that these epithelia function as highly metabolic tissue, expression in thyroid and prostate neoplasia with minimal expression in their normal counterparts casts MMP20‐DSPP cognate pair as potential diagnostic and prognostic markers for neoplasm in these tissues. Retrospective and prospective cohort studies beyond the scope of our study will aim to decipher the prognostic and diagnostic utility in these cancers. Furthermore, we anticipate our data will serve as a major reference and baseline document for studies on the role and significance of MMP20‐DSPP expression in these and other major human epithelial neoplasms.

## CONFLICT OF INTEREST

The authors declare no conflicts of interest.

## AUTHORS’ CONTRIBUTIONS

KUEO: conceived the manuscript; designed all experiment; analysis of results of study; produced initial draft of manuscript; and reviewed final manuscript; JA: contributed to design of immunohistochemistry (IHC) experiment, carried out IHC; analysis of results of study; contributed to draft manuscript; and reviewed final draft of manuscript; KK: contributed to design of IHC experiment, carried out IHC; analysis of results of study; contributed to draft manuscript; GS: contributed to design of in situ proximity ligation assay (iPLA) and immunfluorescence experiment; analysis of results of study; and contributed to draft manuscript; CCA: contributed to design of IHC experiment; analysis of results; draft manuscript.
