# Supplementary material for: Survey of dentin sialophosphoprotein and its cognate matrix metalloproteinase‐20 in human cancers
Source: Cancer Med. 2019 Apr 1;8(5):2167–78. doi: 10.1002/cam4.2117 (PMC6537041; doi:10.1002/cam4.2117)

Supplementary Figures

Supp. Figure-1.

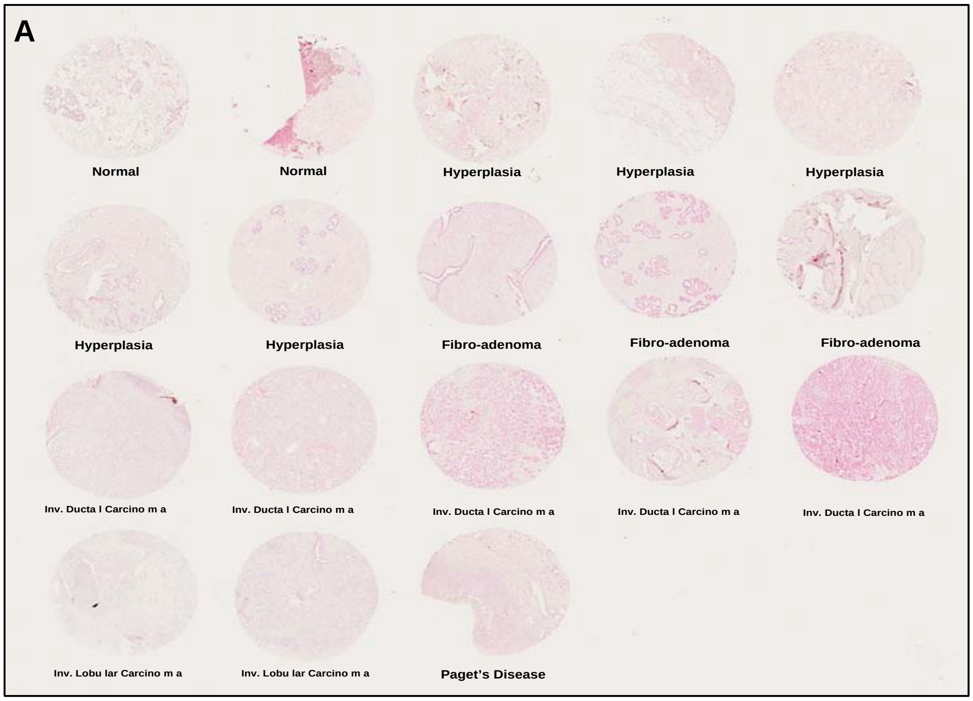

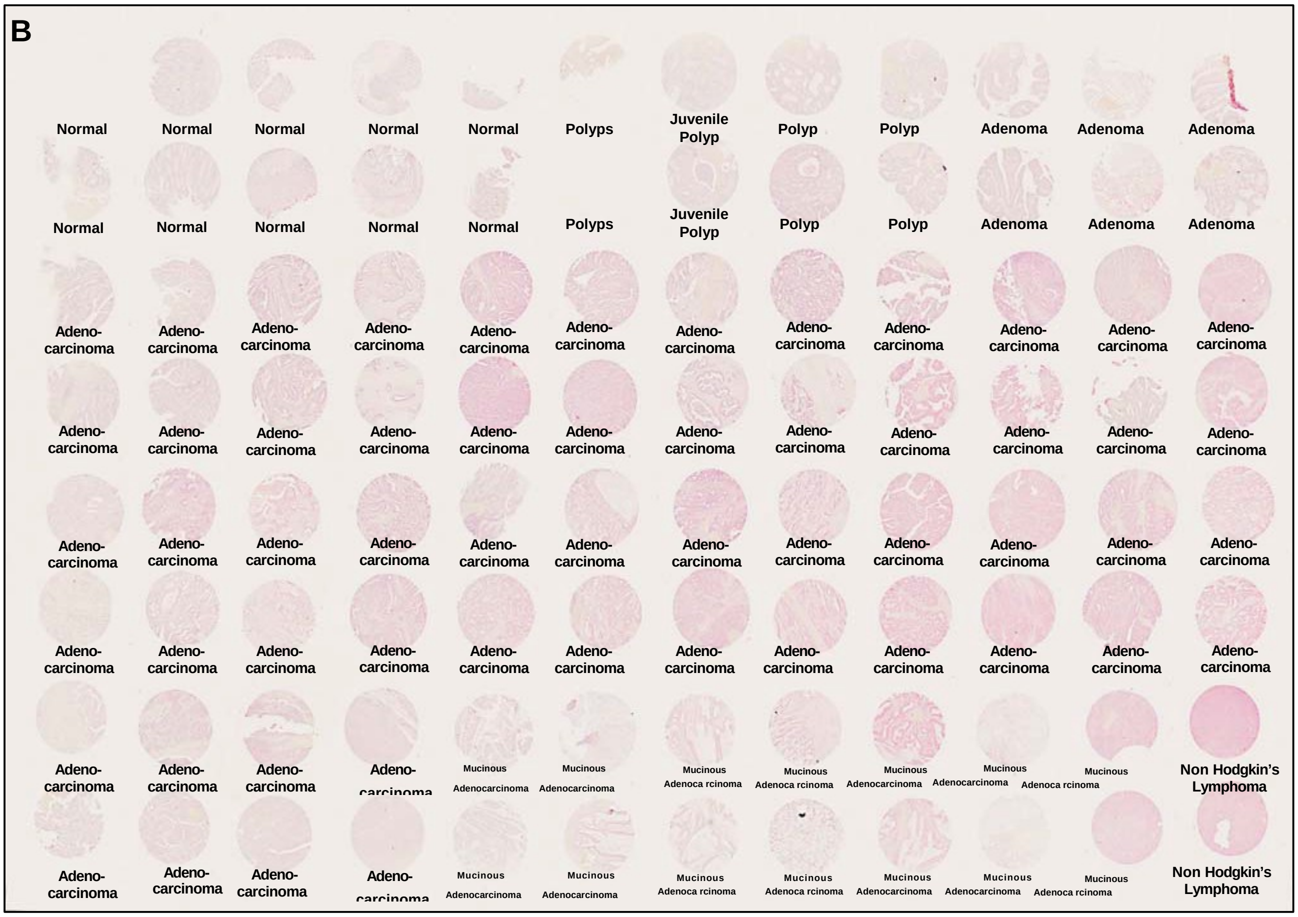

Supp. Figure 1.

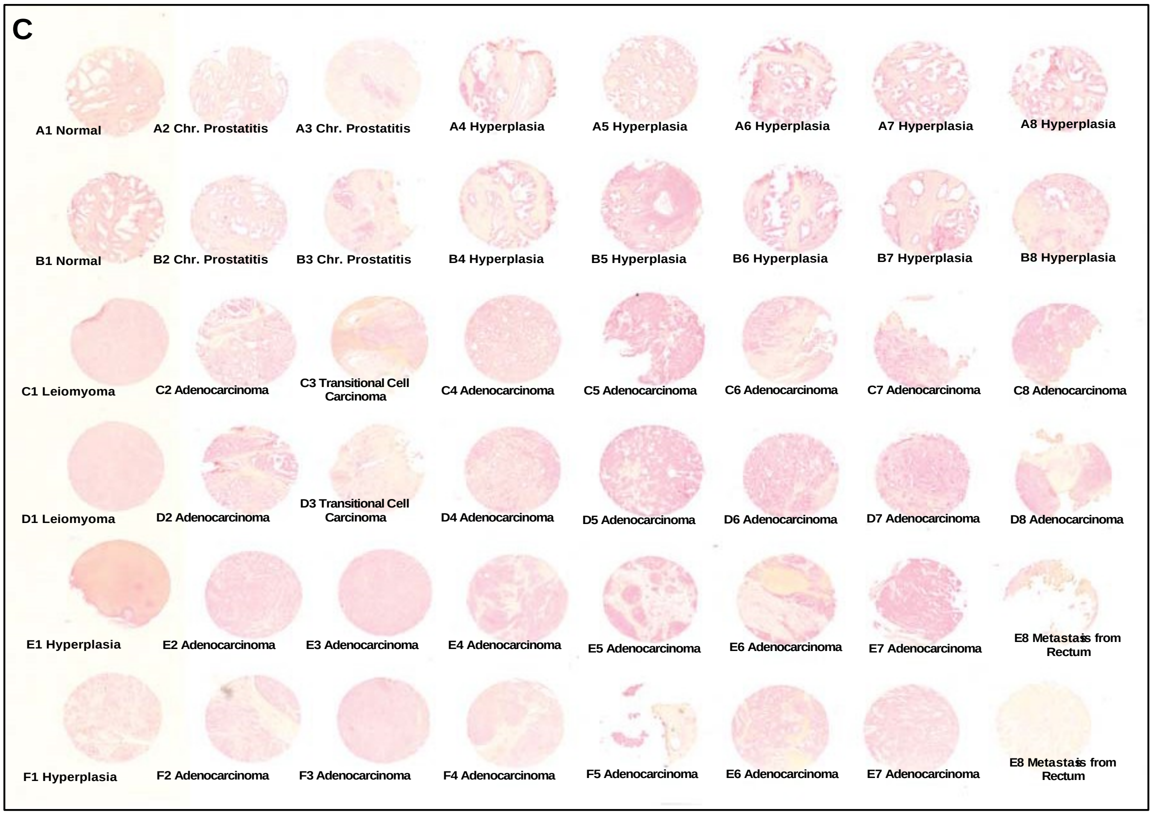

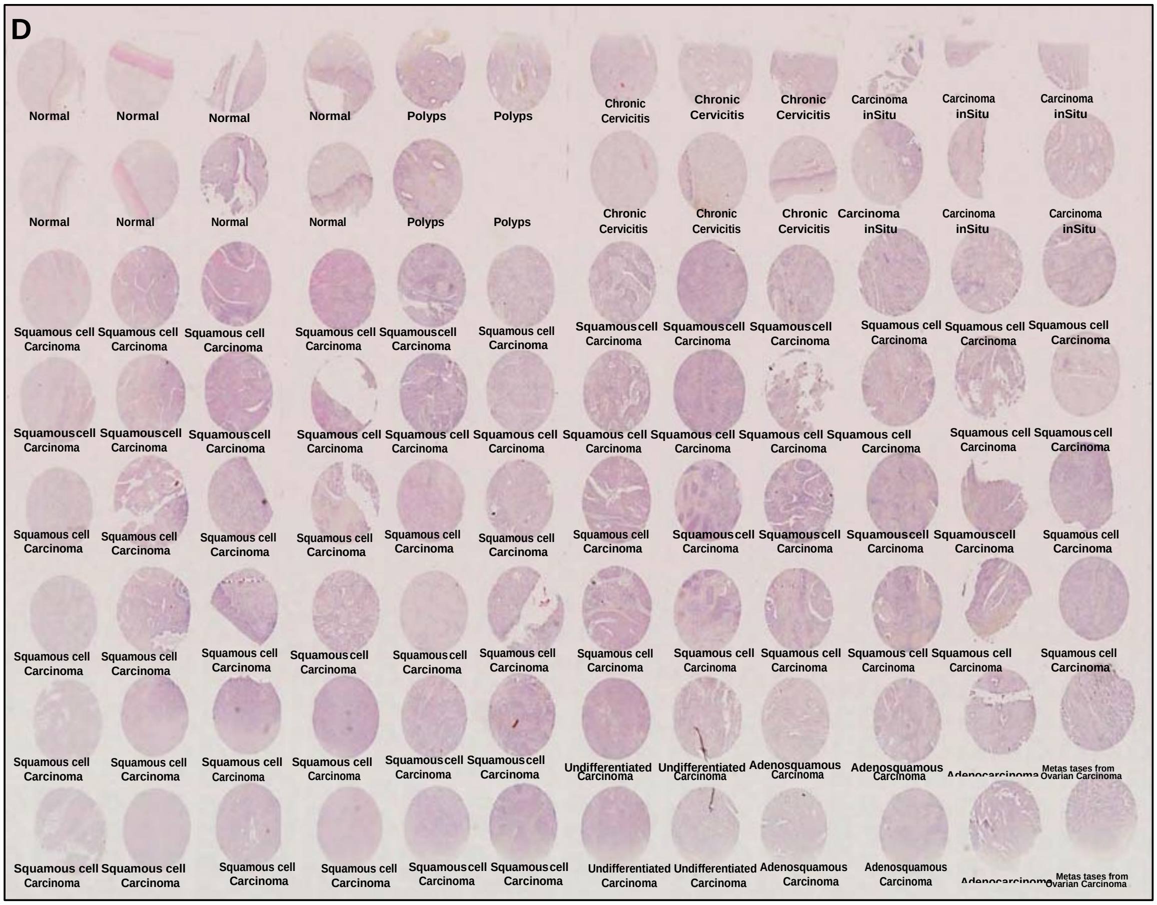

Supp. Figure 1

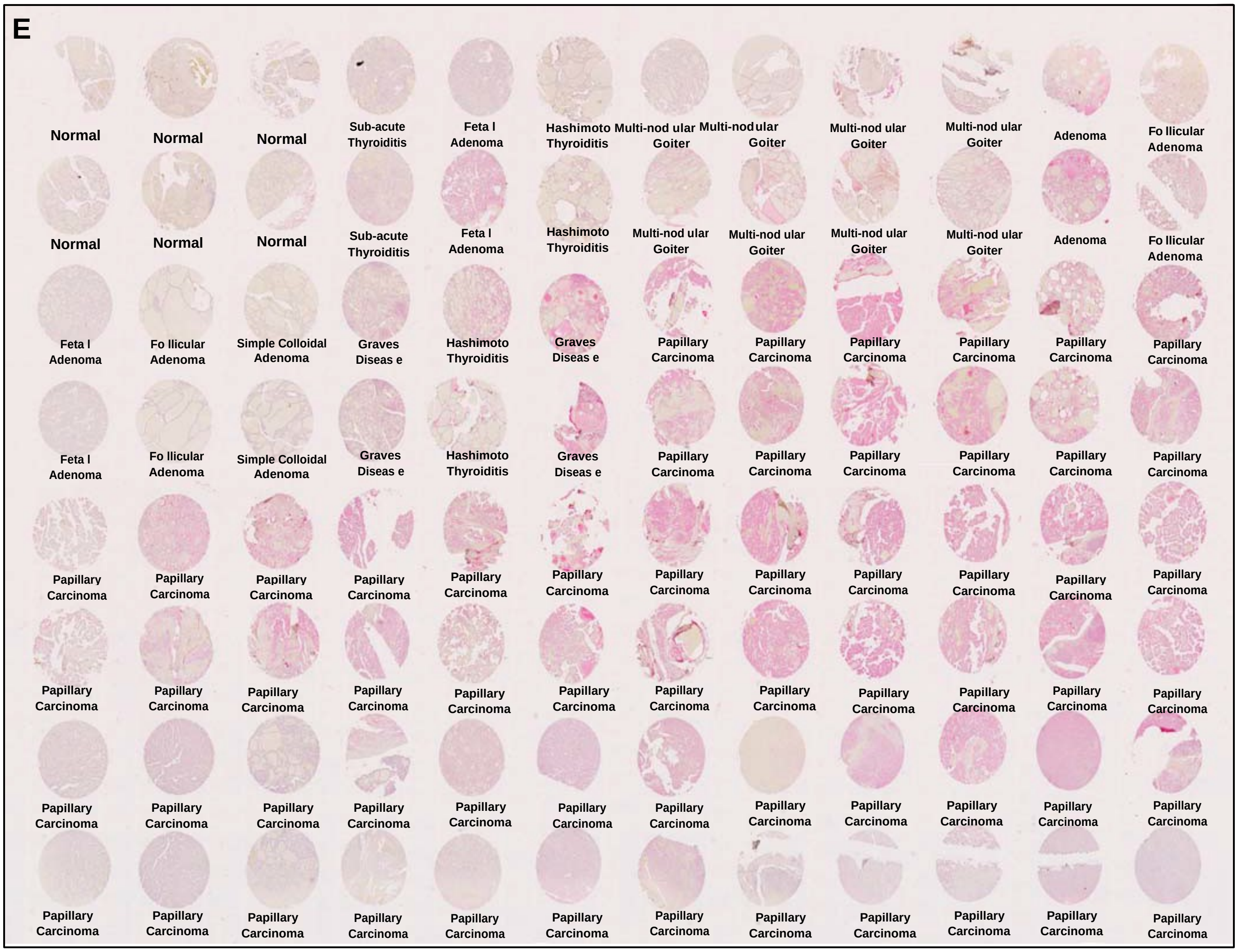

Supp. Figure 1

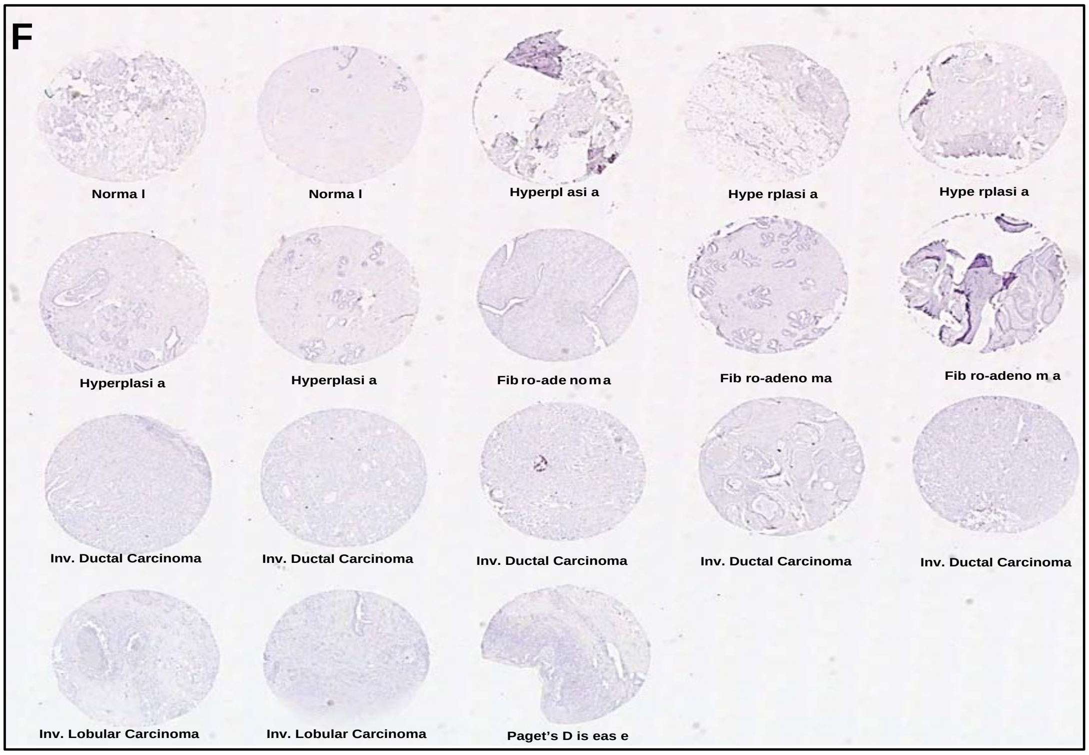

Supp.l Figure-2

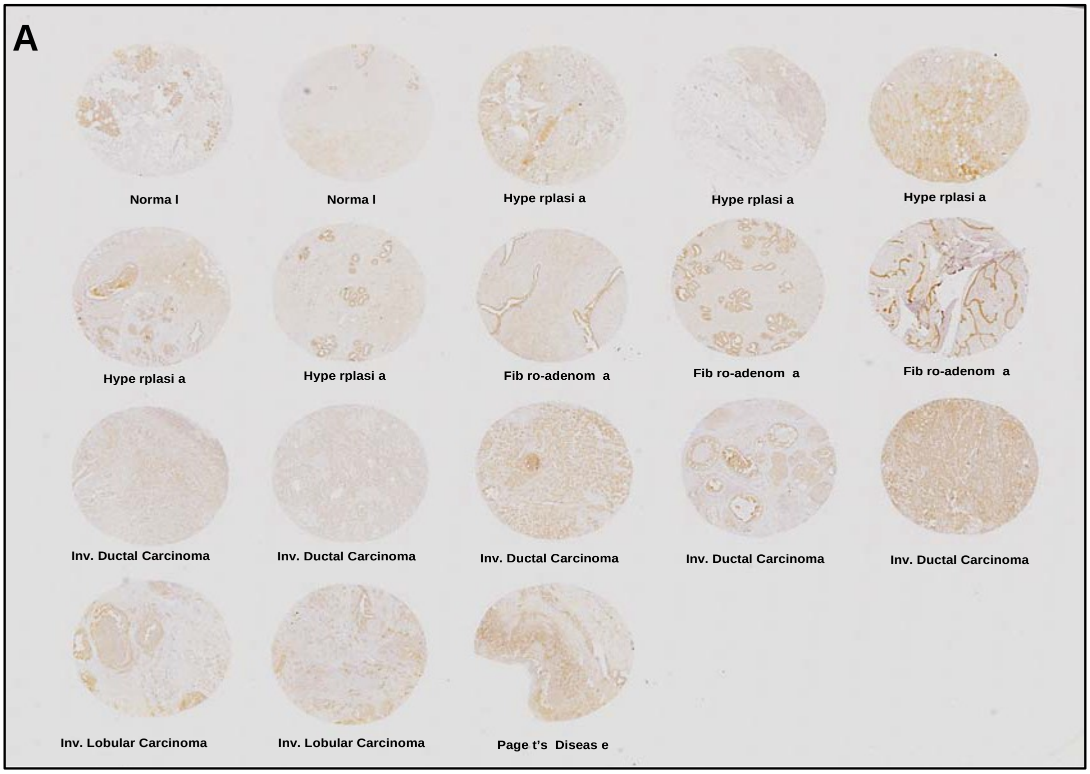

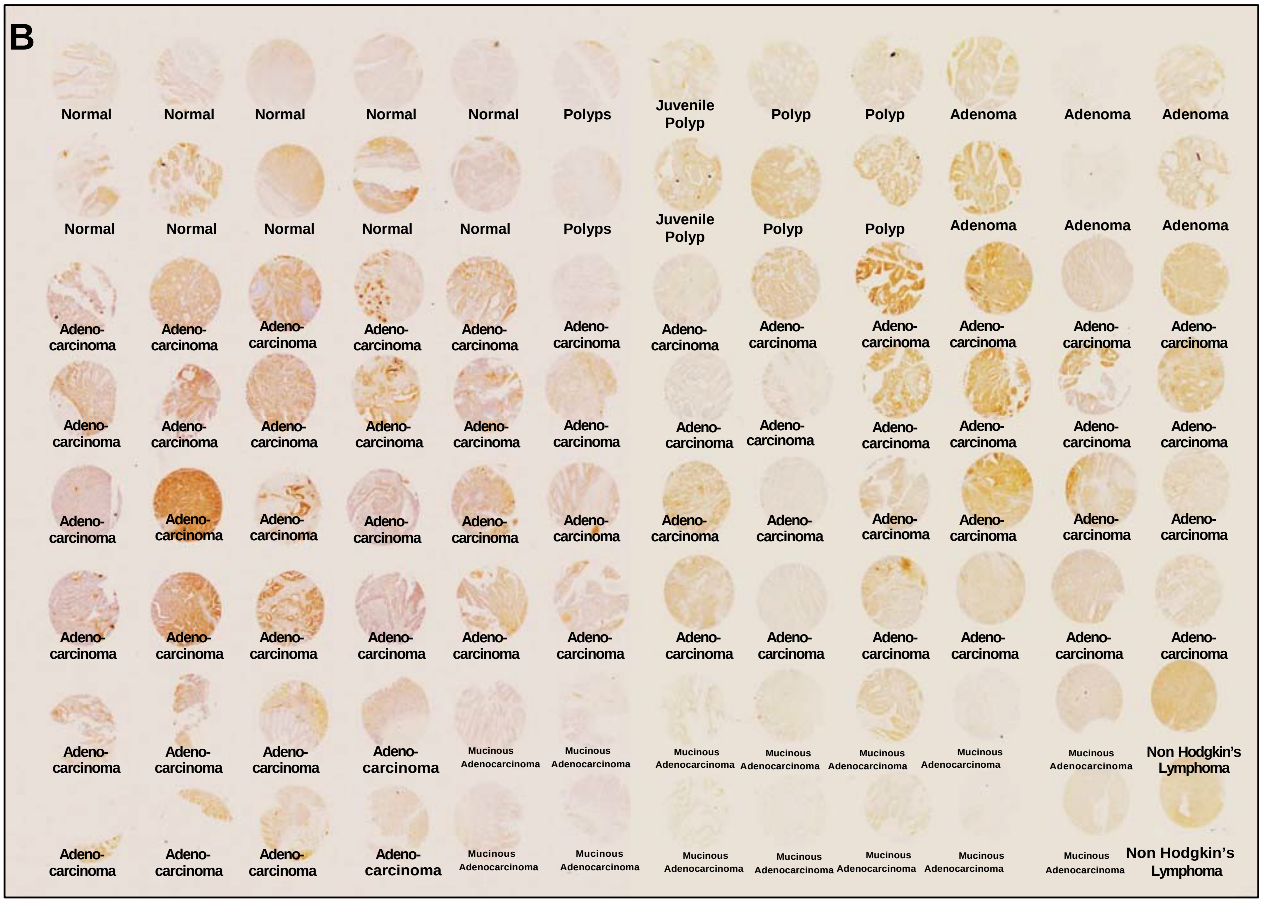

Suppl. Figure 2

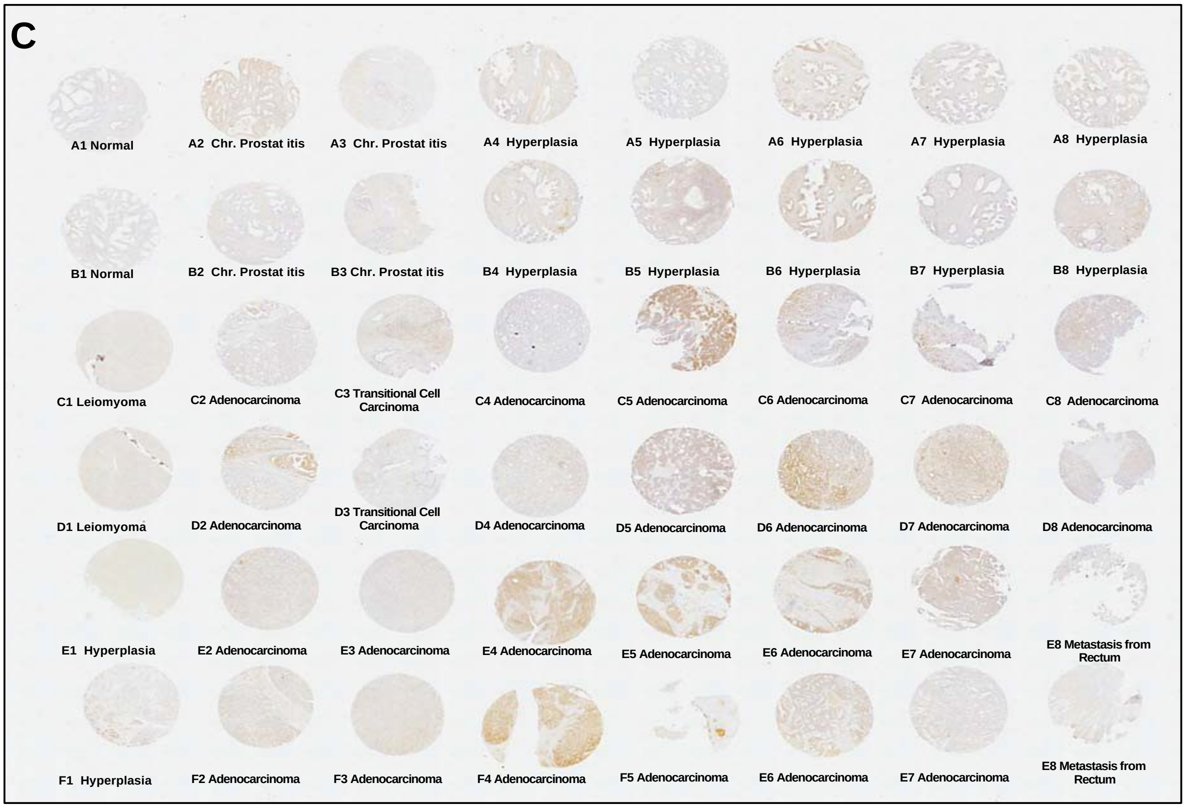

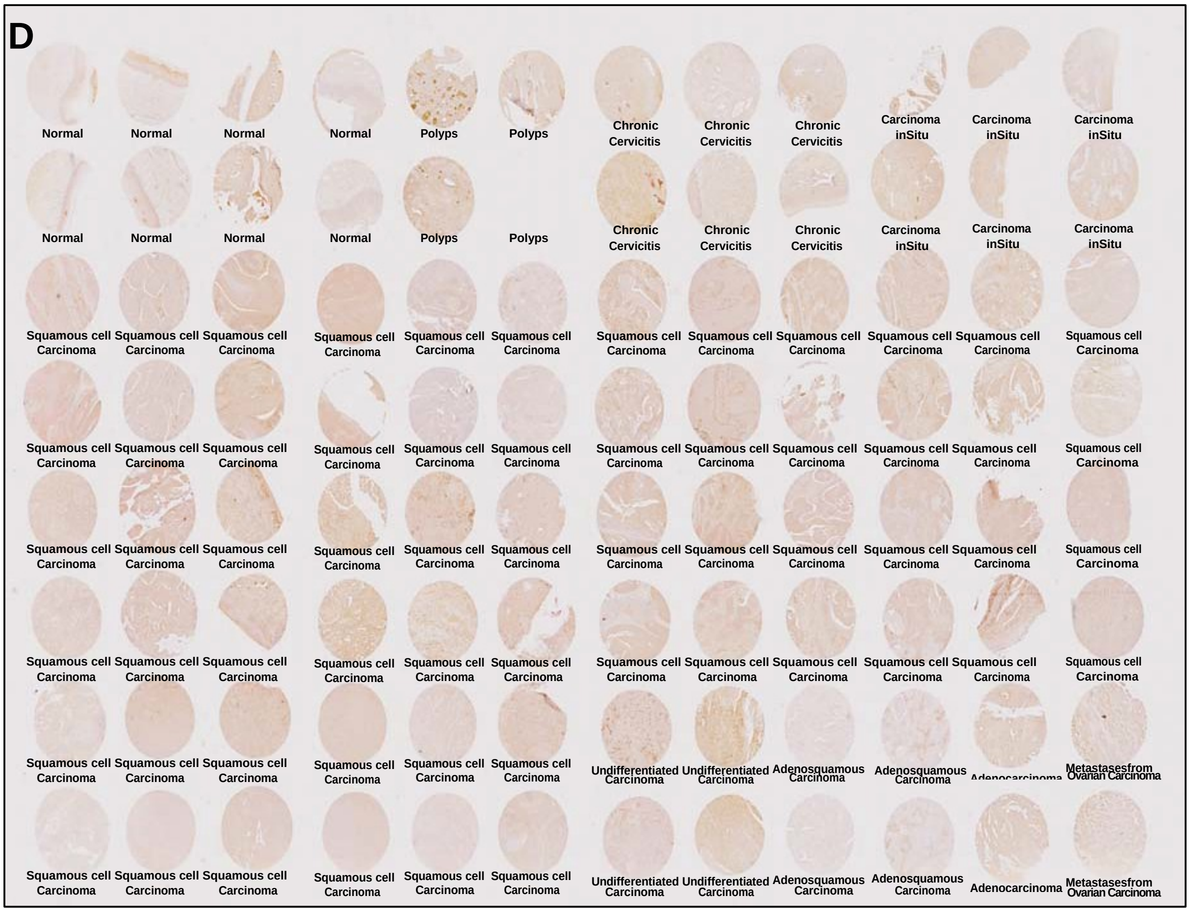

Supp. Figure 2

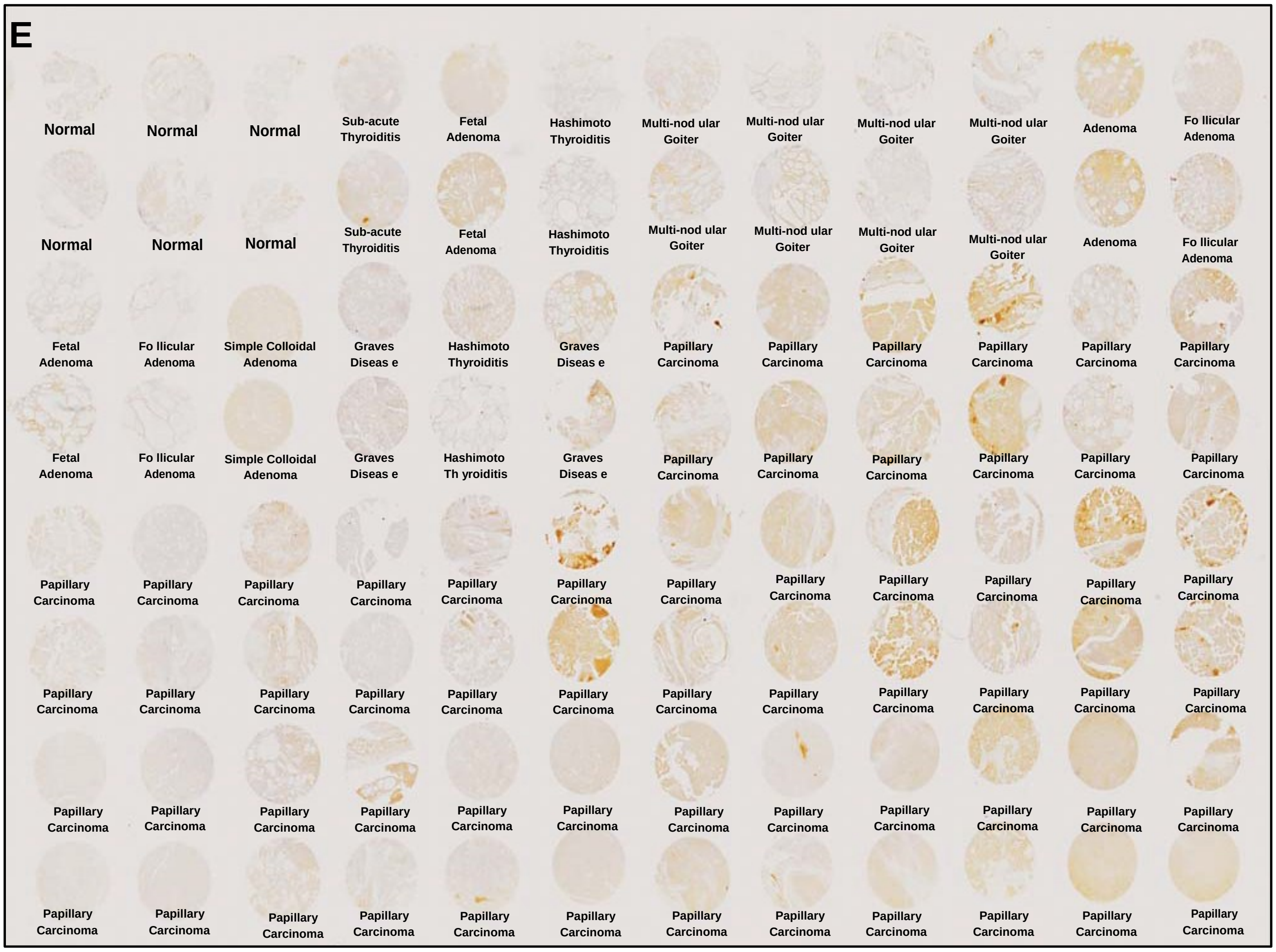

Suppl. Figure 3A

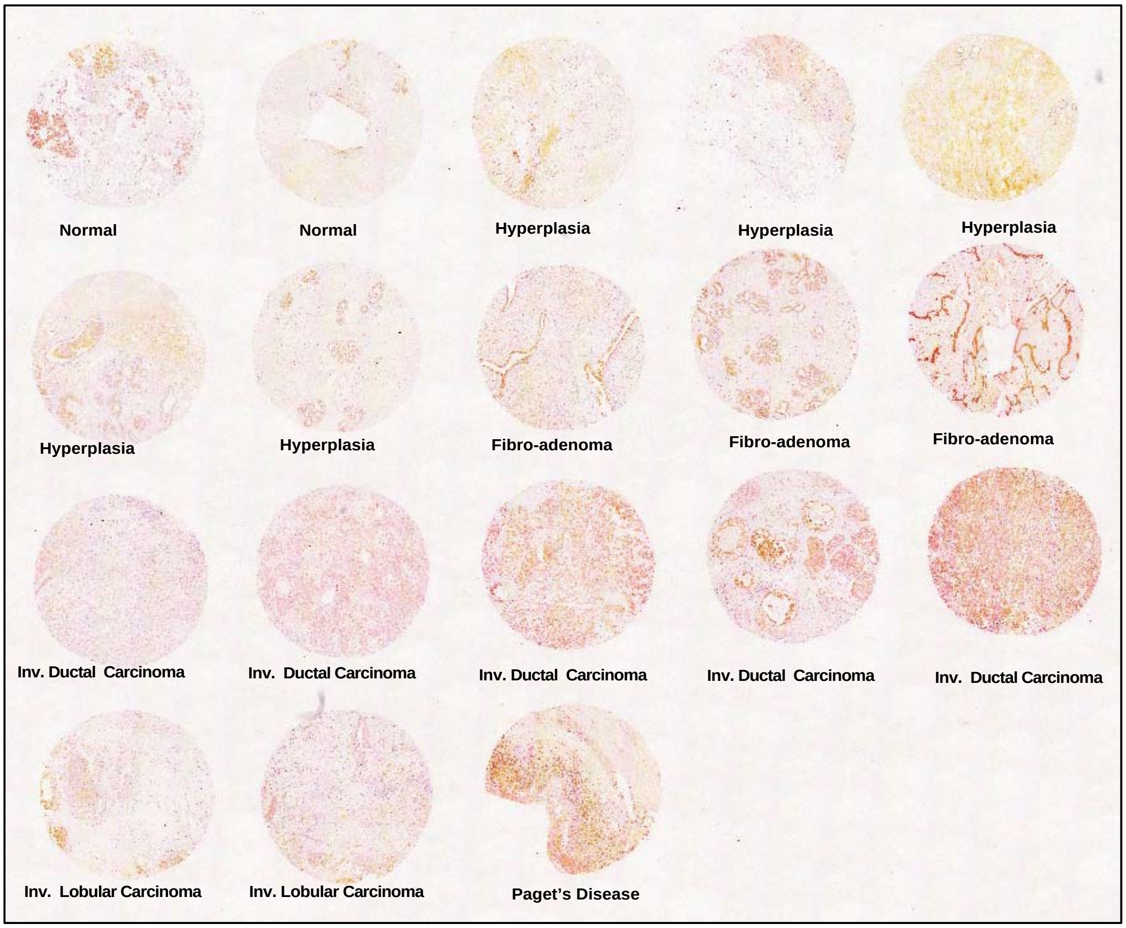

Suppl. Figure 4A

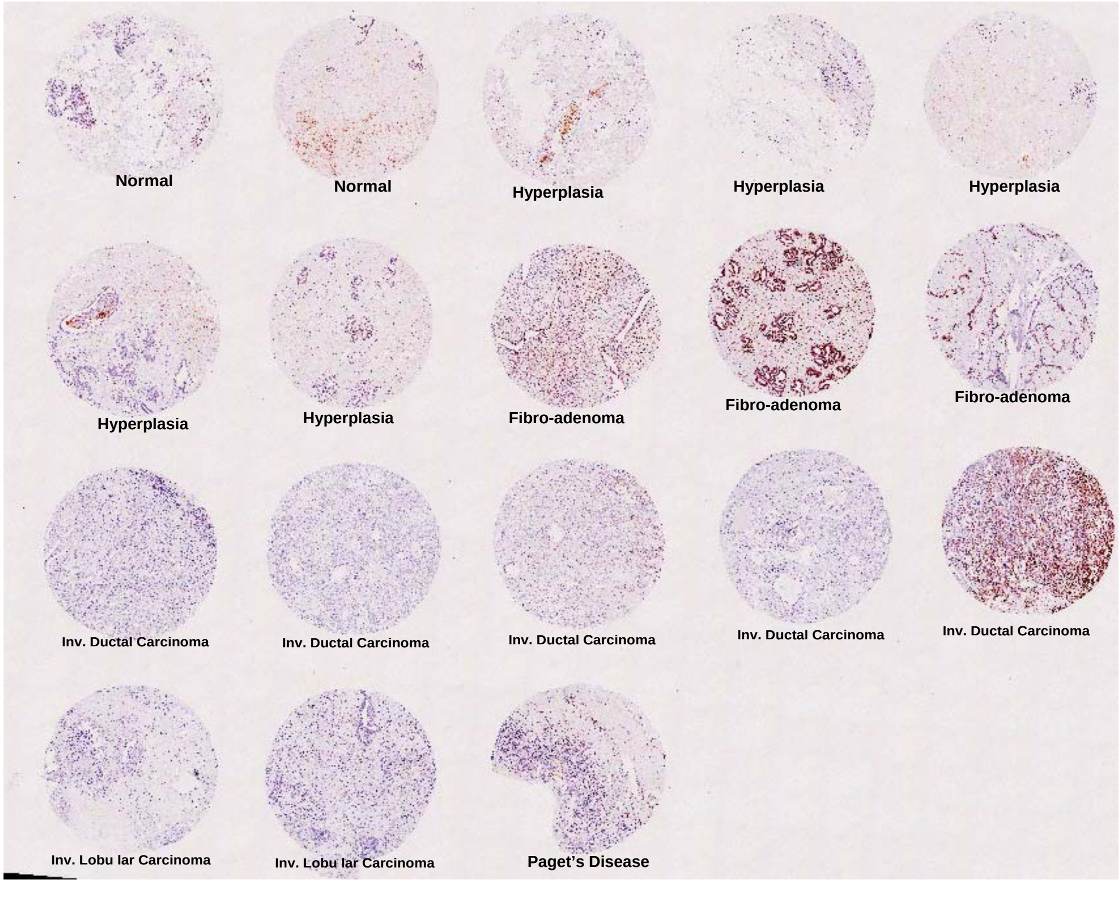

Supp. Figure 3B

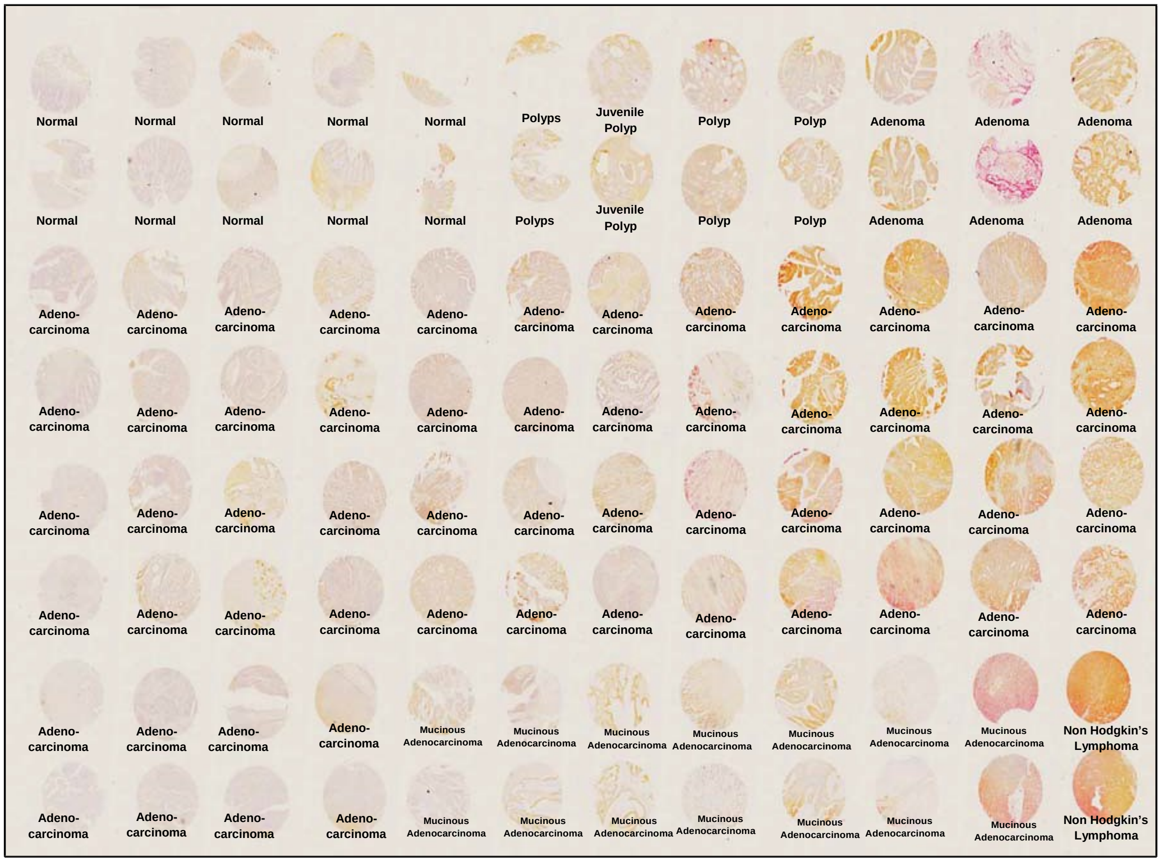

Suppl. Figure 4B.

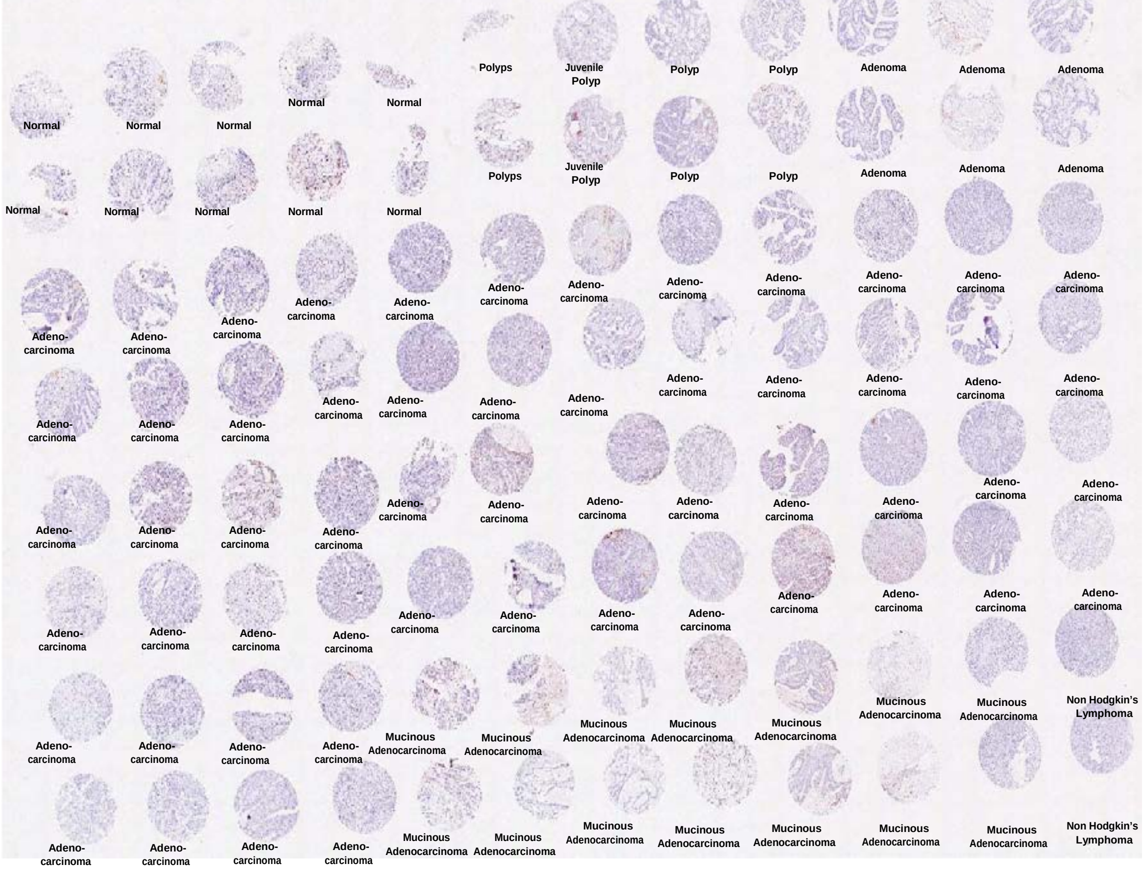

Suppl. Figure 3C

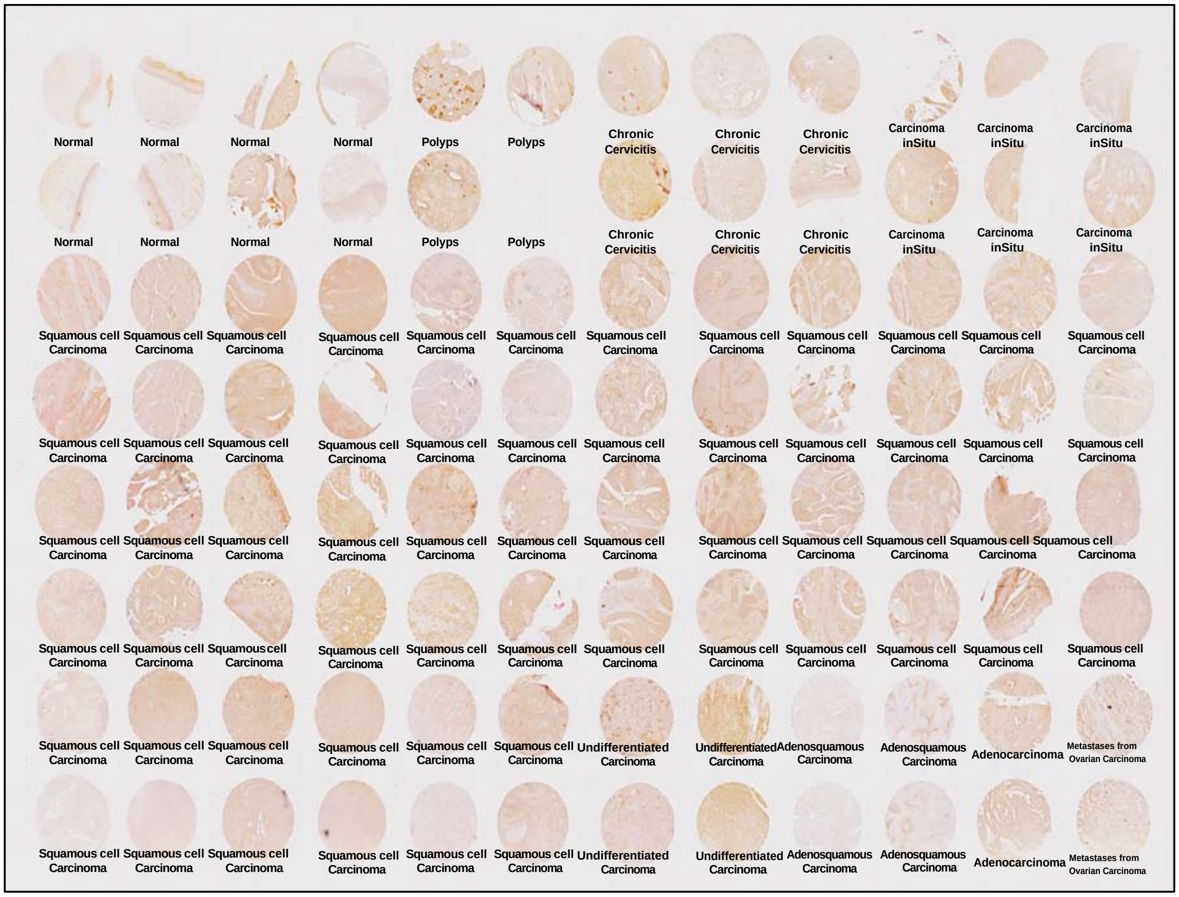

Suppl. Figure 4C

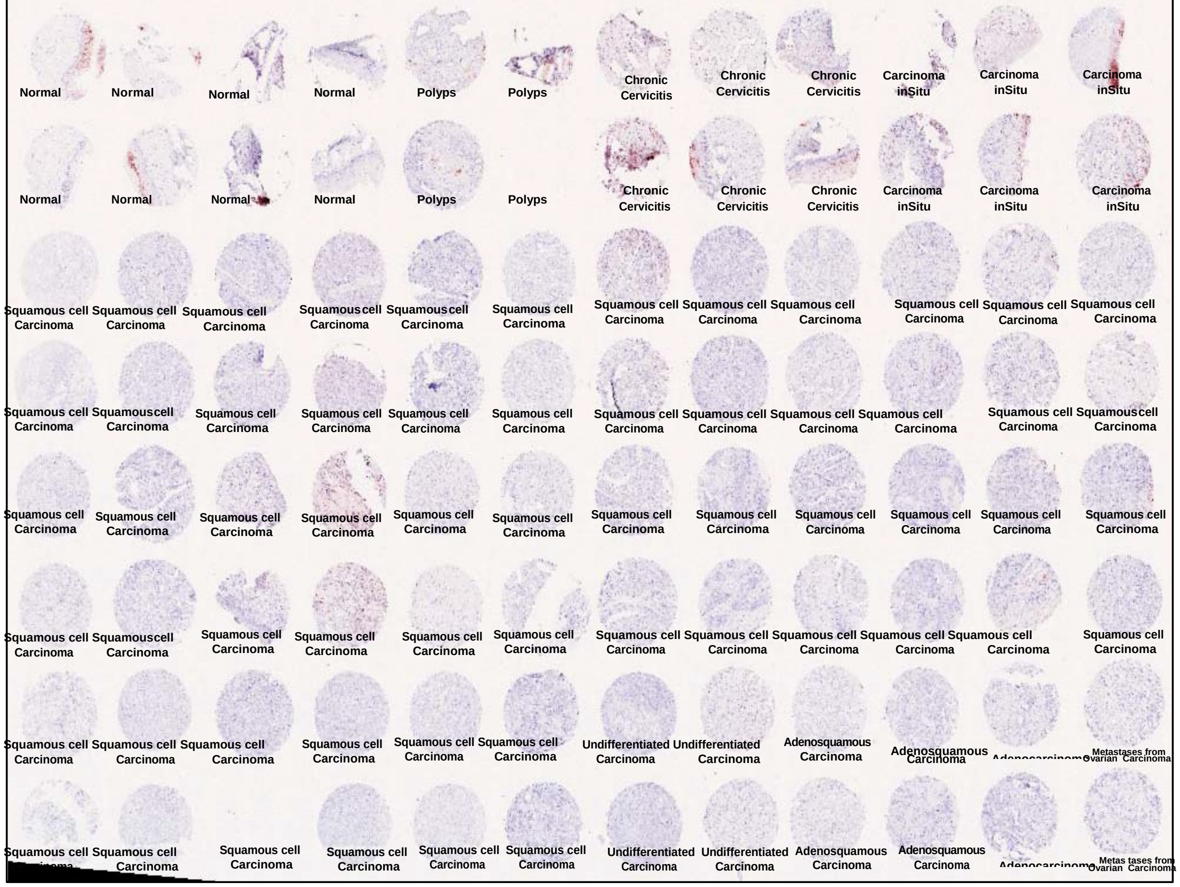

Supplement: Supplementary file 1 [file CAM4-8-2167-s001.pdf]
